# Performance evaluation of the high sensitive troponin I assay on the Atellica IM analyser

**DOI:** 10.11613/BM.2022.020709

**Published:** 2022-06-15

**Authors:** Antonio Buño Soto, Katell Peoc’h, Tommaso Fasano, Jorge Diaz-Garzon, Bernardino González de la Presa, Valerie Chicha-Cattoir, Simone Canovi, Maria Sanz de Pedro, Nayra Rico, Tiphaine Robert, Efrem Bonelli, Pilar Fernández Calle, Aurea Mira, Guillaume Lefevre, Luigi Vecchia, Jose Luis Bedini

**Affiliations:** 1 Laboratory Medicine Department, La Paz University Hospital, Madrid, Spain; 2 Clinical Biochemistry Department, Hospital Beaujon, Clichy, France; 3 Clinical and Metabolic Biochemistry Department, Hospital Bichat, Paris, France; 4 Clinical Chemistry and Endocrinology Laboratory, Department of Diagnostic Imaging and Laboratory Medicine, Hospital Santa Maria Nuova, Reggio Emilia, Italy; 5 Biomedical Diagnostic Center, Hospital Clinic, Barcelona, Spain; 6 Biochemistry and Hormonology Department, Hospital Tenon, Paris, France

**Keywords:** analytical techniques and equipment, high-sensitive troponin I, immunoassay, performance evaluation

## Abstract

**Introduction:**

The Fourth Universal Definition of Myocardial Infarction Global Taskforce recommends the use of high sensitive troponin (hs-Tn) assays in the diagnosis of acute myocardial infarction. We evaluated the analytical performance of the Atellica IM High-sensitivity Troponin I Assay (hs-TnI) (Siemens Healthcare Diagnostics Inc., Tarrytown, USA) and compared its performance to other hs-TnI assays (Siemens Advia Centaur, Dimension Vista, Dimension EXL, and Abbott Architect (Wiesbaden, Germany)) at one or more sites across Europe.

**Materials and methods:**

Precision, detection limit, linearity, method comparison, and interference studies were performed according to Clinical and Laboratory Standards Institute protocols. Values in 40 healthy individuals were compared to the manufacturer’s cut-offs. Sample turnaround time (TAT) was examined.

**Results:**

Imprecision repeatability CVs were 1.1–4.7% and within-lab imprecision were 1.8–7.6% (10.0–25,000 ng/L). The limit of blank (LoB), detection (LoD), and quantitation (LoQ) aligned with the manufacturer’s values of 0.5 ng/L, 1.6 ng/L, and 2.5 ng/L, respectively. Passing-Bablok regression demonstrated good correlations between Atellica IM analyser with other systems; some minor deviations were observed. All results in healthy volunteers fell below the 99th percentile URL, and greater than 50% of each sex demonstrated values above the LoD. No interference was observed for biotin (≤ 1500 µg/L), but a slight bias at 5.0 g/L haemoglobin and 50 ng/L Tn was observed. TAT from was fast (mean time = 10.9 minutes) and reproducible (6%CV).

**Conclusions:**

Real-world analytical and TAT performance of the hs-TnI assay on the Atellica IM analyser make this assay fit for routine use in clinical laboratories.

## Introduction

The Fourth Universal Definition of Myocardial Infarction (UDMI) Global Task Force recommends the use of high sensitive cardiac troponin (hs-Tn) assays to identify myocardial injury from any cause, including acute myocardial infarction (AMI) (*1*). Myocardial injury is defined as Tn concentrations above the 99th percentile upper reference limit (URL) of a healthy reference population, and AMI is defined as “evidence of myocardial necrosis in a clinical setting consistent with myocardial ischemia and the presence of a significant rise and/or fall of Tn with at least one value above the 99th percentile URL”, with one other hallmark of AMI (*1*). Concerning AMI diagnosis, hs-Tn assays can detect rising Tn concentrations earlier than contemporary assays following myocardial injury (within one hour *vs.* three to six hours) (*2*). The improved precision of hs-Tn assays at low concentrations allows for shorter time intervals between serial measurements to detect a rising or falling pattern and enables the use of rapid rule-out/rule-in diagnostic strategies (*2*-*5*). For healthcare professionals to effectively implement measurement of Tn with hs-Tn assays, recommendations on the use of hs-Tn assays were published in 2018 by the Academy of the American Association for Clinical Chemistry (AACC) and the The Inernational Federation of Clinical Chemistry and Laboratory Medicine (IFCC) Task Force on the Clinical Application of Cardiac Bio-Markers and other experts (*6*, *7*). Preanalytical and analytical issues, time to reporting results from either sample receipt or collection (important for rapid algorithms), and other laboratory-related issues were highlighted. Quality control measures at lower analytical detection limits, sex-specific 99th percentile URL values, and determination of reference intervals were recommended. An assay was designated as high-sensitive if it demonstrated a coefficient of variation (CV) of ≤ 10% at the sex-specific 99th percentile URL and detected Tn values greater than or equal to the limit of detection (LoD) in at least 50% of healthy populations of each sex. Because hs-Tn assays are not standardized, cut-off values for rule-out, rule-in, significant delta changes, and the 99th percentile URL must be determined for each assay and sex; and the latter cut-off value may vary depending on the criteria used to select the reference population (*6*, *8*-*11*).

The goal of this study was to evaluate the analytical performance of the high sensitive troponin I (hs-TnI) assay on the Atellica IM analyser (Siemens Healthcare Diagnostics Inc., Tarrytown, USA) following the recommendations of the AACC/IFCC Task Force and other experts (*6*, *7*).

## Materials and methods

The analytical performance of the hs-TnI assay on the Atellica IM analyser was performed at four sites across Europe from December 2017 to March 2018 (Site A: Barcelona, Spain. Site B: Clichy, France; Paris, France. Site C: La Paz, Madrid, Spain. Site D: Reggio Emilia, Italy).

### Materials

Precision samples included Liquichek Cardiac Markers Plus Control, (PTNIH-L1 to L3 and PTNIH-LT) (BioRad Laboratories), and low-end Tn lithium-heparin plasma pools PTNIH-SP (SP1 to SP6) designed to have recovery 0.20x (SP1, SP3, and SP5) and 0.50x (SP2, SP4, and SP6) the manufacturer’s 99th percentile URL. A different lot of the Bio-Rad control material PTNIH-LT was tested at Site D. For detection capability, a low zero calibrator was used the for limit of blank (LoB) verification. Serum pools were used for the limit of detection (LoD) and limit of quantitation (LoQ) verification. The target concentration determined for the LoD sample prior to testing was 1.6 ng/L (LoD of the manufacturer). For LoQ, serum samples with low hs-TnI assay concentrations around 2.5 ng/L were pooled to create two separate samples of approximately 2.5 ng/L with a volume of 5 mL. From each pooled sample, five 1 mL aliquots were frozen at - 20 ^o^C or - 80 ^o^C (a total of ten aliquots). For linearity, Atellica IM analyser master curve material (human TnI complex in a human serum matrix) was assessed as unknowns. When the highest concentrations exceeded the assay range, they were excluded from the analysis. For interference studies, target concentrations for TnI were 50 ng/L and 100 ng/L in serum (for biotin), and lithium heparin plasma (for haemolysis) samples. Biotin stock was created using biotin in the form of powder (Sigma Chemical Company, St. Louis, USA) dissolved in 0.1 M NaOH. Before using it as a spiker to create the samples, this biotin stock was diluted to 1:10 concentration (40,000 µg/L biotin), using 0.1 M NaOH as diluent. Control (unspiked) samples contained 0.1 M NaOH. Haemolysed samples were prepared by spiking with haemoglobin (Hb) (Red Cell Lysate, 162 g/L) (The Binding Site, San Diego, USA). Control (unspiked) samples contained phosphate buffered saline.

### Subjects

Method comparison studies were performed with frozen patient lithium heparin plasma samples collected at each of the four sites from routine Tn testing. For values in healthy individuals, serum TnI concentrations were measured in samples stored at – 80 ºC for two years from males (N = 20; age range: 24–57 years) and females (N = 20; age range 24–58 years) who had no history of relevant heart disease or co-morbidities associated with myocardial damage. Exclusion criteria were as follows: occult diabetes mellitus (serum glucose ≥ 7 mmol/L), kidney disease (glomerular filtration rate estimated with the Chronic Kidney Disease Epidemiology Collaboration (CKD-EPI) equation < 60 mL/min/1.73 m^2^), and impaired cardiac function (serum NT-proBNP > 125 pg/mL) (*12*). Within a two-year follow-up, the subjects did not have related heart disease history or co-morbidities associated with myocardial damage, including diabetes mellitus. When necessary, informed consent was obtained from individuals included in this study. The research related to human use complied with all the relevant national regulations, institutional policies, the Helsinki Declaration and was approved by the authors’ institutional review boards or equivalent committees.

### Methods

#### High sensitive troponin immunoassay on the Atellica IM analyser

The Atellica IM High-Sensitivity Troponin I (TnIH) assay is a three-site sandwich immunoassay based on acridinium ester chemiluminescent technology. The assay employs biotinylated mouse and sheep monoclonal antibodies pre-bound to the solid phase *via* streptavidin to avoid biotin interference. The Lite Reagent contains a proprietary acridinium ester and one recombinant anti-human TnI sheep monoclonal Fab fragment bound to bovine serum albumin for chemiluminescent detection. Each antibody/antibody fragment recognizes a different TnI epitope. A direct relationship is observed between the amount of TnI in the sample and relative light units detected. The risk of interference by heterophilic antibodies is reduced due to the use of Fab fragment instead of the entire IgG (*13*). According to the manufacturer, there was no statistical difference between the 99th percentile URL values based on sample type (serum or lithium heparin plasma); values for males, females, and combined are 53 ng/L, 34 ng/L, 45 ng/L for lithium heparin plasma (*13*). A summary of the characteristics of this and other assays used in this study is presented in Table 1. Assays were performed according to manufacturer instructions.

**Table 1:** Summary of assay characteristics according to manufacturer

**Manufacturer**	**Assay**	**Platform**	**Principle of test**	**Measuring range** **(ng/L)**	**Time to first result (minutes)**
Siemens Healthcare Diagnostics Inc.	High-Sensitivity Troponin I (TnIH)	Atellica IM analyser	3-site CLIA	2.5–25,000	10
Siemens Healthcare Diagnostics Inc.	High-Sensitivity Troponin I (TnIH)	Advia Centaur	Same as for hs-TnI assay on Atellica IM analyser above	2.5-25,000	18
Siemens Healthcare Diagnostics Inc.	High-Sensitivity Troponin I (TnIH)	Dimension Vista	Homogeneous sandwich CLIA based on LOC^I®^ technology.	3.0–25,000	10
Siemens Healthcare Diagnostics Inc.	High-Sensitivity Troponin I (TnIH)	Dimension EXL	Same as for hs-TnI assay on Dimension Vista	4.0–25,000	10
Abbott	STAT High Sensitive Troponin-1	Architect	2-step immunoassay using CMIA technology.	10–50,000	16
CLIA – Chemiluminescent immunoassay technology. LOCI – Luminescent oxygen channeling assay. CMIA – Chemiluminescent microparticle immunoassay. STAT – Short turn around testing. hs-TnI – high sensitive troponin I.					

#### Imprecision

Repeatability and within-lab (total) imprecision studies were performed according to Clinical and Laboratory Standards Institute (CLSI) Document EP15-A3 and AACC/IFCC TF-CB recommendations and expressed as mean, standard deviation (SD), and the coefficient of variation (CV%) (*6*, *14*). Each sample was run in duplicate, two runs *per* day, over ten days for a total of 20 runs and 40 replicates. Results were compared with those of the manufacturer.

#### Detection capability

The LoB, LoD, and LoQ studies were performed as described in CLSI Document EP17-A2 at Sites A and C (*15*). The LoB corresponds to the highest measurement result that is likely to be observed for a blank sample. The LoD corresponds to the lowest concentration that can be detected with a probability of 95%. The LoQ corresponds to the lowest amount of analyte in a sample with a within-laboratory precision of ≤ 20%. Two samples for LoB and two samples for LoD were processed in the same run with four replicates each day for three days, for a total of 24 measurements each. One freshly thawed aliquot from each of the two LoQ pools was analysed as one run *per* day, five replicates *per* run, for five days, for a total of 25 replicates (total of 50 replicates). The limit of blank would be verified if at least 86% of samples were ≤ LoB reported by the manufacturer (0.5 ng/L), and LoD would be demonstrated if at least 86% of samples were ≥ LoB. For LoQ, greater than 86% of samples had to be within the acceptable range of 2.0–3.0 ng/L (*i.e.,* to verify LoQ *per* CLSI EP17-A2, the acceptability window around 2.5 ng/L (LoQ according to the manufacturer) is calculated using the allowable error from the manufacturer instructions for use (which is 20%). Thus, all values which fall between 2.0 and 3.0 ng/L verify the LoQ claim.

#### Linearity

Linearity studies were performed according to CLSI Document EP06-A (Sites B and C) (*16*). Five concentration levels of linearity material (0.0, 53.8, 135.0, 1980.0, and 20,620.0 ng/L) with three replicates *per* level were processed in a randomized order.

#### Method comparison

Method comparison analysis was performed according to CLSI Document EP09-A3 at the four sites (*17*). Instead of running samples in duplicate, samples were run in singlicate. Comparisons were obtained between hs-TnI assays on the Atellica IM analyser with Advia Centaur, Dimension EXL, Dimension Vista, and Architect STAT systems.

#### Values in healthy individuals

Tn values in healthy individuals were compared with the cut-offs according to the manufacturer (Site D).

#### Biotin and haemolysis interference

Interference studies were performed according to CLSI Document EP7-A2 (Site C) (*18*). Target concentrations for TnI were 50 ng/L and 100 ng/L (including those near the overall 99th percentile URL of 45 ng/L). Serum samples were spiked with biotin stock to achieve biotin concentrations of 100 µg/L and 1500 µg/L. Spiked and control samples were run in duplicate. Lithium heparin plasma samples were spiked with Hb to achieve Hb concentrations of 1.5 g/L and 5.0 g/L. Spiked and control samples were run in duplicate.

#### Turnaround time (TAT)

A typical three-hour peak period of the day was recreated in the Atellica Solution for 1561 immunoassay test requests and 13 hs-TnI short turn around testing (STAT) tests (Site C). Intra-analyser TAT was measured from the time of barcode reading to the result delivery. Subsequently, under routine operating conditions, intra-analyser and total (from sample registration to results delivery) TATs were determined for 1462 additional test requests and 13 hs-TnI STAT tests (Site C). The reaction time of the assay is 10 minutes (min). Total TAT from sample registration to results reporting was also obtained for 10 hs-TnI assay samples run concurrently with 750 immunoassays (total tests = 10,200) (Site A).

### Statistical analysis

Precision and linearity analyses were performed with a software program termed “Eval Tools” (version 1.3 and 3.3) (Siemens Healthcare Diagnostics Inc., Tarrytown, USA) which utilizes the analysis procedures outlined by CLSI (*14*, *16*, *19*). Linearity was evaluated using weighted linear regression. Method comparison (Passing-Bablok regression) analysis was performed with Analyze-It add-in, version 4.10 for Microsoft Excel according to CLSI procedures (*17*).

## Results

Repeatability CVs across four sites were 1.1–4.7%, and within-lab imprecision CVs were 1.8–7.6%, for concentrations ranging from 10 to 25,000 ng/L (Table 2). The results are comparable to those of the manufacturer (*13*). Each level tested was < 10% at each of the four sites, including levels much lower than the 99th percentile URLs. Here, the within-lab imprecision CV was 2.5% at 40 ng/L (a concentration near to the female cut-off, *i.e.,* 34 ng/L), and across four sites, concentrations ranging from 10.7 to 11.9 ng/L demonstrated within-lab imprecision CVs of 5.5 to 7.6%.

**Table 2:** Repeatability and within-lab (total) imprecision for the hs-TnI assay on the Atellica IM analyser at different sites

**Site**	**Sample type**	**Mean Conc. (ng/L)**	**Repeatability**		**Within-Lab (total)**	
			**SD**	**CV(%)**	**SD**	**CV(%)**
A	SP1 SP2 LT L1 L2	11.9 26.4 99.2 260.9 5863.9	0.5 0.7 1.6 4.3 66.9	4.1 2.6 1.6 1.7 1.1	0.9 1.5 4.4 11.9 186.9	7.6 5.5 4.4 4.6 3.2
B	SP1 SP2 LT L1 L2	10.7 25.0 95.0 252.8 5717.0	0.4 0.7 1.5 4.4 67.0	3.6 2.9 1.6 1.7 1.2	0.7 0.9 3.8 10.9 154.9	6.8 3.4 4.0 4.3 2.7
C	SP1 SP2 LT L1 L2	11.0 25.2 93.0 245.6 5577.7	0.4 0.6 1.8 5.5 78.8	3.6 2.2 2.0 2.2 1.4	0.6 0.8 3.0 7.6 118.7	5.5 3.0 3.3 3.1 2.1
D	SP1 SP2 L1 L2 L3	11.2 25.4 40.3 3684.1 19,228.1	0.5 1.0 0.7 57.4 447.5	4.7 3.8 1.7 1.6 2.3	0.8 1.0 1.0 67.6 675.3	6.7 3.9 2.5 1.8 3.5
hs-TnI – high sensitive troponin I. SD – standard deviation. CV – coefficient of variation. Conc. – concentration.						

The limit of blank and LoD samples met the stated criteria. For LoB, 96% of low zero calibrator samples were ≤ LoB reported by the manufacturer (0.5 ng/L), and 100% of LoD samples were ≥ LoB) (Supplementary Tables 1 and 2). For LoQ the criterion was also met (Supplementary Table 3). Specifically, 23/25 = 0.92 of results verified for Sample 1 and 22/25 = 0.88 of results verified for Sample 2, in total 45/50 = 0.90. According to the CLSI document, for 50 total results, at least 0.88 of the results must verify. Since we have 0.90, LoQ is verified. The LoQ is also verified if each sample is looked at separately. In this case, at least 0.86 of the results need to verify.

The linearity study involved the evaluation of predicted *versus* observed values for five concentrations of linearity material. The assay demonstrated linear results by weighted fit, yielding the following equations: y = 0.96x + 0.21, range 1.6–21,138.4 ng/L at one site and y = 1.02x + 1.71, range 1.8–21,153.6 ng/L at another site.

Passing-Bablok regression results are shown in Table 3. Regression analysis yielded a linear equation for each comparison and 95% confidence interval (95% CI) for intercept and slope. Methods were considered interchangeable if 95% CI for intercept included the value of zero and 95% CI for slope included the value of one. Constant and proportional error were identified where 95% CI for intercept and slope did not include the stated values, respectively (*20*). Good correlation was obtained between the Atellica IM analyser and the other Siemens Healthineers assays (r ranged between 0.984 and 0.999). Some minor constant and/or proportional deviations were observed. Site A demonstrated proportional error (95% CI for slope did not include the value of one), Site B had constant error (95% CI for intercept did not include the value of zero), Sites C and D demonstrated both deviations. However, at three sites, the 95% CI of the slope was very close to one (1.00–1.03, 1.04–1.08, 1.03–1.06). Thus, the results could be considered acceptable. Good correlation was also obtained between the hs-TnI assays on the Atellica IM analyser and Dimension Vista and Dimension EXL systems. Interchangeability was found for the Dimension EXL, but a constant deviation was observed for Dimension Vista. The correlation study between the Atellica IM TnIH assay and the Architect assay was good (r = 0.96), but a constant deviation was observed, and some scatter was seen at high concentrations.

**Table 3:** Correlation and Passing-Bablok regression results for hs-TnI assays on the Atellica IM 1600 Analyser and established systems

**Site**	**Comparative hs-cTn assay**	**N**	**Measuring range** **(ng/L)** **(Atellica IM)**	**Slope**	**95% CI of slope**	**Intercept** **(ng/L)**	**95% CI of intercept**	**Correlation coefficient**
A	Advia Centaur XP^‡^	39	11.84–18,397	0.88	0.86–0.90	0.77	-0.33–2.61	0.997
B	Advia Centaur XP*	87	2.21–15,989	1.02	1.00–1.03	0.98	0.67–1.41	0.999
C	Advia Centaur XP^§^	117	2.19–7,223	1.06	1.04–1.08	0.87	0.66–1.10	0.999
D	Advia Centaur XP^§^	72	1.53–20,212	1.05	1.03–1.06	-2.31	-2.70–(-1.63)	0.999
B	Architect STAT*	91	1.34–15,989	0.99	0.88–1.14	1.46	0.28–2.71	0.964
C	Dimension Vista^†^	121	1.49–7,223	1.04	1.00–1.07	-1.37	-1.63–(-0.76)	0.984
A	Dimension EXL*	40	11.84–19,566	0.97	0.93–1.01	-0.24	-6.12–9.48	0.998
CI – coefficient of variation. hs-Tn – high sensitive troponin. *Intercept 95% CI includes value zero and slope 95% CI includes value one; ^†^Intercept 95% CI does not include value zero and slope 95% CI includes value one; ^‡^Intercept 95% CI includes value zero and slope 95% CI does not include value one; ^§^Intercept 95% CI does not include value zero and slope 95% CI does not include value one								

The TnI concentrations in 20 male and 20 female healthy volunteers were below the stated sex-specific and overall 99th percentile URL cut-offs. Males (16/20), females (11/20), and overall (27/40) had values that fell between the stated LoD 1.6 ng/L and the 99th percentile URLs. Thus, greater than 50% of each sex and subjects overall had measurable values above the LoD.

No interference was observed for biotin ≤ 1,500 µg/L. A slight bias at 5.0 g/L Hb and 50 ng/L TnI was observed (Table 4).

**Table 4:** Interference of biotin and haemolysis on the hs-assay on the Atellica IM analyser

**Sample** **Biotin conc.** **(µg/L)**	**hs-Tn** **Target conc.** **(ng/L)**	**hs-Tn** **Average**	**Bias (%)**	**Sample** Hb* conc. **(g/L)**	**hs-Tn** **Target conc.** **(ng/L)**	**hs-Tn** **Average**	**Bias (%)**
Control (100)	50	50.90	/	Control (1.5)	50	46.86	/
Spike (100)	50	50.38	-1.03	Spike (1.5)	50	48.21	2.81
Control (1500)	50	48.49	/	Control (5.0)	50	49.79	/
Spike (1500)	50	48.80	0.65	Spike (5.0)	50	44.70	-11.39
Control (100)	100	99.19	/	Control (1.5)	100	96.31	/
Spike (100)	100	97.76	-1.46	Spike (1.5)	100	95.86	-0.46
Control (1500)	100	93.99	/	Control (5.0)	100	96.43	/
Spike (1500)	100	92.455	-1.67	Spike (5.0)	100	94.375	-2.18
*Hb – Haemoglobin. hs-Tn – high sensitive troponin. conc. – concentration. Recovery was expressed in %.							

The average Atellica IM intra-analyser TAT for the hs-TnI assay run while performing 1651 routine tests was 10.9 min (*i.e.,* 10 min and 9/10 x 60 = 54 seconds) from the time of the first barcode read to the result (hs-TnI range, 10.5–12.7 min; CV = 6%; N = 13). The TAT was predictable with minimal impact on the throughput of routine samples. These findings were confirmed for 1462 additional routine tests and 13 hs-TnI STAT tests, and the mean total TAT from sample registration to results delivery of 44.4 min (Table 5) was confirmed at another site for ten hs-TnI samples.

**Table 5:** Results of the Atellica IM 1600 turnaround time (TAT) for the hs-TnI assay during testing of 1461 routine and 13 hs-TnI STAT samples

	**Total TAT**	**TAT** **from registration** to entrance in IOM*	**TAT** **from IOM to centrifuge (connected)**	**TAT** **from entrance in IOM to sample aspiration in Atellica IM analyser**	**TAT** **in Atellica IM analyser (inside the analyser): from bar code reading to result**
Mean (min.)	44.4	6.8	19.3	27.2	10.7
SD (min.)	12.4	5.5	3.0	8.0	0.4
P90 (min.)	63.1	14.0	23.6	38.4	11.2
CV (%)	28	80	16	29	4
Minimum	18.2	0.0	13.5	7.0	10.1
Maximum	74.7	29.5	29.8	44.8	15.8
*IOM – input/output module from the APTIO Automation Solution (Siemens Healthcare Diagnostics Inc., Tarrytown, USA) used with the Atellica IM analyser. TAT – Turnaround time. SD – standard deviation. CV – coefficient of variation. Min – minutes. STAT – short turnaround time. hs-TnI – high sensitive troponin I. P90 – 90th percentile.					

## Discussion

In the emergency department (ED), ordering a Tn test constitutes an emergency with significant challenges. Results must be provided quickly and accurately with optimal sensitivity and specificity without interferences that might cause false-positive and false-negative results. High sensitive troponin assays have been developed to address these challenges.

This study demonstrates several key findings. First, imprecision aligned with the values of the manufacturer and was considered acceptable. Our results are consistent with the findings of others (*9*). For comparison, a total CV of 10% was found at 5.6 ng/L and a CV of 5% at 9 ng/L on the Architect hs-TnI assay (*21*). Second, low values were achieved for LoB, LoD, and LoQ that agreed with values reported by the manufacturer. Guidelines have recommended the LoD, LoQ (20% CV), or other low values as cut-offs for rule-out of AMI in patients presenting to the ED with suspicious AMI (*6*). The LoQ 20%CV value of 2.5 ng/L (rounded to the whole number 3.0 ng/L) for this hs-TnI assay has been used clinically with rapid protocols by others in Europe and the USA with very high negative predictive values for AMI (*4*, *6*, *22*, *23*). The advantages of hs-TnI assays, besides improved analytical performance, are earlier AMI diagnosis in the ED, decreased crowding in the ED, and lower costs.

Overall good agreement between the hs-TnI assays on the Atellica IM analyser and Advia Centaur system was not surprising because they use the same reagent formulations. However, we could not demonstrate interchangeability between both methods. Interchangeability was demonstrated for Dimension EXL, but not for Dimension Vista or for Architect. (The Dimension Vista and Dimension EXL hs-TnI assays use the same three antibodies in the assay as the Atellica IM and Advia Centaur assays but in a different configuration). If a laboratory has two different analysers, it must decide whether to consider both methods fully interchangeable or not based on the interchangeability data and other analytical performance.

In this study, 40 healthy adults had Tn concentrations below the 99th percentile URL of the manufacturer and of an independent study (AACC USB) (*8*). The latter reported slightly lower cut-offs (males: 44 ng/L; females: 26 ng/L; overall: 38 ng/L) which were attributed to different selection criteria of subjects (*10*). Also here, the second criterion for a high sensitive assay was supported in that greater than 50% of males, females, and overall had values greater than the LoD. These results are consistent with those of two independent studies with much larger populations of more than 300 subjects for each sex - the HIGH-US study and the AACC USB study (*8*, *9*). Although samples from the AACC USB were used for both studies, each study was performed in a different facility with different selection criteria (*24*).

Biotin is found in many nutritional supplements and is part of the treatment for several conditions (metabolic or neurological disorders). New high-dose biotin formulations may cause interferences with laboratory immunoassays that use biotin-streptavidin. The incidence is growing, with several reports describing falsely high or low results leading to misdiagnosis. It has been reported that megadoses taken up to 300 mg/day may lead to serum concentrations of about 1160 µg/L. Here, biotin interference was considered insignificant up to 1500 µg/L at 50 and 100 ng/L cTnI. This result was not surprising because preformed biotin-streptavidin was included in the assay design to mitigate biotin interference. A ≤ 10% change up to 3500 µg/L was reported by the manufacturer and confirmed recently in an independent study (*25*). Haemolysis interference was considered insignificant up to 5 g/L Hb (at 100 ng/L TnI) but slightly exceeded the acceptance limit at 5 g/L Hb (at 50 ng/L cTn). The manufacturer reported ≤ 10% change up to 5 g/L Hb and this was confirmed in a recent independent publication (*25*). The reason why our results differ from those two reports warrants investigation.

Current guidelines recommend hs-Tn assays for use with rapid diagnostic algorithms (*2*-*5*). Thus, generating results with the fastest time possible is mandatory for the clinical laboratory, especially for STAT analysis (*6*, *7*). Ideally, Tn results should be reported within 60 min of when a sample is received, and better yet from when the sample was collected (*6*). Here, we verified the manufacturer’s mean intra-analyser TAT; met the stated goal from when the sample was received; and achieved a mean TAT within one hour from sample registration to results reporting. The TAT inside the analyser was consistently low (10.7 min with a CV of 4%) with wider differences observed in other parts of the automation process. We continue to improve on the stated goal from the time of sample collection.

This study had limitations. Sample pools had to be prepared for very low concentrations because commercial material did not exist; this was challenging. Therefore, we could not calculate accuracy at the lower medical decision levels (*26*, *27*). The 99th percentile URLs were not determined here but previously by others; thus, we verified if values in 40 healthy individuals fell within the stated cut-offs. Some experiments used serum and other lithium heparin plasma samples because they can be used interchangeably (*13*, *28*); however, lithium heparin plasma is more appropriate for the emergency room because serum may increase the risk of hypercoagulability, microclots, fibrin, or particulate matter in some patients, interfering with results (*29*, *30*). Finally, not all evaluations were performed at all four sites.

In conclusion, the hs-TnI assay on the Atellica IM analyser demonstrated acceptable imprecision, correlated well with other Siemens Healthcare Diagnostics assays, and had good and predictable STAT TAT with minimal effect on throughput (*6*, *7*). Detection capability was consistent with that reported by the manufacturer. Values (> 50%) in healthy individuals fell within the cut-offs of the manufacturer. Taken together, our results on imprecision and in healthy subjects provide additional support that the hs-TnI assay fulfills both requirements for the performance of a high sensitive assay. Thus, the assay is deemed fit for routine use in clinical practice.
